# The Association between HbA1c, Fasting Glucose, 1-Hour Glucose and 2-Hour Glucose during an Oral Glucose Tolerance Test and Cardiovascular Disease in Individuals with Elevated Risk for Diabetes

**DOI:** 10.1371/journal.pone.0109506

**Published:** 2014-10-06

**Authors:** Marcus Lind, Jaakko Tuomilehto, Matti Uusitupa, Olle Nerman, Johan Eriksson, Pirjo Ilanne-Parikka, Sirkka Keinänen-Kiukaanniemi, Markku Peltonen, Aldina Pivodic, Jaana Lindström

**Affiliations:** 1 Institute of Medicine, Sahlgrenska Academy University of Gothenburg, Gothenburg, Sweden; 2 Center for Vascular Prevention, Danube University Krems, Krems, Austria; 3 National Institute for Health and Welfare, Helsinki, Finland; 4 King Abdulizaz University, Jeddah, Saudi Arabia; 5 Institute of Public Health and Clinical Nutrition, University of Eastern Finland, and Research Unit, Kuopio University Hospital, Kuopio, Finland; 6 Chalmers University of Technology, University of Gothenburg, Gothenburg, Sweden; 7 National Institute for Health and Welfare, Department of Chronic Disease Prevention, Helsinki, Finland; 8 University of Helsinki, Department of General Practice and Primary Health Care, Finland Vasa Central Hospital, Vasa, Finland; 9 Folkhälsan Research Centre, Helsinki, Finland; 10 Unit of General Practice, Helsinki University Central Hospital, Helsinki, Finland; 11 Finnish Diabetes Association, Tampere, Finland; 12 Institute of Health Sciences, University of Oulu, Oulu, Finland; 13 Unit of General Practice, Oulu University Hospital, Oulu, Finland; 14 Statistiska konsultgruppen, Gothenburg, Sweden; Heinrich-Heine University, Faculty of Medicine, Germany

## Abstract

**Objective:**

To determine the association between HbA1c, fasting plasma glucose (FPG), 1-hour (1 hPG) and 2-hour (2 hPG) glucose after an oral glucose tolerance test (OGTT) and cardiovascular disease in individuals with elevated risk for diabetes.

**Design:**

We studied the relationship between baseline, updated mean and updated (last) value of HbA1c, FPG, 1 hPG and 2 hPG after an oral 75 g glucose tolerance test (OGTT) and acute CVD events in 504 individuals with impaired glucose tolerance (IGT) at baseline enrolled in the Finnish Diabetes Prevention Study.

**Setting:**

Follow-up of clinical trial.

**Participants:**

504 individuals with IGT were followed with yearly evaluations with OGTT, FPG and HbA1c.

**Main Outcome Measure:**

Relative risk of CVD.

**Results:**

Over a median follow-up of 9.0 years 34 (6.7%) participants had a CVD event, which increased to 52 (10.3%) over a median follow-up of 13.0 years when including events that occurred among participants following a diagnosis of diabetes. Updated mean HbA1c, 1 hPG and 2 hPG, HR per 1 unit SD of 1.57 (95% CI 1.16 to 2.11), p = 0.0032, 1.51 (1.03 to 2.23), p = 0.036 and 1.60 (1.10 to 2.34), p = 0.014, respectively, but not FPG (p = 0.11), were related to CVD. In analyses of the last value prior to the CVD event the same three glycaemic measurements were associated with the CVD events, with HRs per 1 unit SD of 1.45 (1.06 to 1.98), p = 0.020, 1.55 (1.04 to 2.29), p = 0.030 and 2.19 (1.51 to 3.18), p<0.0001, respectively but only 2 hPG remained significant in pairwise comparisons. Including the follow-up period after diabetes onset updated 2 hPG (p = 0.003) but not updated mean HbA1c (p = 0.08) was related to CVD.

**Conclusions and Relevance:**

Current 2 hPG level in people with IGT is associated with increased risk of CVD. This supports its use in screening for prediabetes and monitoring glycaemic levels of people with prediabetes.

## Introduction

The global prevalence of diabetes is increasing rapidly, and in 2030 over 500 million individuals are expected to suffer from diabetes, mostly type 2 (T2D) [1]. T2D is a progressive disease, both in terms of glycaemia and its resulting complications. In order to halt diabetes progression in high-risk individuals and to avoid the likely burden of future diabetic complications, management of individuals with prediabetes, characterized as having above-normal blood glucose levels, but not meeting the diagnostic criteria of diabetes needs to be focused upon [2]. The landmark Finnish Diabetes Prevention Study (DPS) showed that intensive lifestyle intervention among individuals with impaired glucose tolerance (IGT) effectively prevented progression from IGT to diabetes [3]. This benefit has been subsequently confirmed by others [4], [5].

The collaborative Diabetes in Europe: Classification and Diagnostic Criteria (DECODE) study confirmed that asymptomatic hyperglycaemia is associated with an increased risk of premature mortality and cardiovascular disease (CVD, [6]). In particular, there was a graded relationship between mortality and 2-hour post-challenge plasma glucose (2 hPG). However, few studies have examined the effects of intensive glycaemic management in individuals with prediabetes in order to prevent CVD, mortality or other complications due to hyperglycaemia, and results have been equivocal [7], [8]. In a recent systematic review, the relationships between 2-h plasma glucose (2 hPG) and fasting plasma glucose (FPG) and risk of future CVD and mortality were shown to be relatively weak [2]. However, these studies only examined baseline FPG and 2 hPG measurements, thereby information on glycaemic control during follow-up (i.e., when the events actually occurred), may have weakened the associations. Moreover, it is unclear whether prediabetes *per se* and/or the development of diabetes during a later point in time is relevant to the association between IFG, IGT and CVD events, as well as relevant confounders including physical activity, which were generally missing in previous studies [2].

To address this gap in knowledge we studied data from the original DPS trial, including FPG, 1 hPG, 2 hPG and HbA1c in relation to the development of CVD [3], as well as extended follow-up data that included regularly recorded glucose measurements but not continued intensive lifestyle intervention.

## Methods

### Participants

The DPS design and study participants have been described in detail elsewhere (NCT00518167, clinicaltrials.gov) [3]. In brief, the DPS examined the extent to which lifestyle intervention can prevent or delay the future onset of diabetes in individuals with IGT. The original randomized phase of the trial ended in 2001, and an extended follow-up phase was performed afterward. An OGTT was completed on each annual visit with measurements of FPG, 1 hPG, 2 hPG and HbA1c during both phases. Information on medication use and CVD events were obtained by linking trial data to the Finnish national drug prescription register, death register and hospital discharge register.

Among the original cohort of 522 individuals, 17 did not consent to record linkage, leaving 505 individuals available for the current analysis. One individual was excluded due to lack of information on the timing of diabetes diagnosis during follow-up.

The study protocol was approved by the Ethics Committee of the National Public Health Institute in Helsinki, Finland and all the study participants gave written informed consent.

### Procedures

An incident CVD event was defined as a composite endpoint of the first myocardial infarction, stroke, unstable angina, coronary artery bypass graft or percutaneous coronary intervention (PCI). ICD-8 and 9 codes 410, 4110, 431, 433, 4330A, 4331A, 4339A, 434, 4340A, 4341A, 4349A, 436 and ICD-10 codes I200, I21, I22, I61, I63, I64 were used to obtain event data.

We analysed the relation between the composite CVD events and baseline, updated values, and updated mean FPG, 1 hPG, 2 hPG and HbA1c measurements. The updated value was defined as the most recent recording of a glucose measurement prior to an event or the end of follow-up for an individual. The updated mean is defined as the mean value at every recording of a new glucose measurement and includes all follow-up recordings prior to the event or end of follow-up [8]. Updated mean HbA1c has been shown to have greater predictive power for diabetic complications than the use of baseline variables alone in prospective studies in patients with types 1 and 2 diabetes [9]. To be included in the analyses, glucose measures had to exist at baseline or year 1. Missing baseline values were extrapolated from year 1 data, concerning 11 values of HbA1c and 61 values of 1 hPG. In analyses of FPG and the 2 hPG value in relation to CVD, all 504 individuals had information at baseline and were included in the analysis. The number of individuals with information at baseline or at year 1 included in the 1 hPG and HbA1c analyses was 498 and 502, respectively. In main analyses, individuals were followed until the first CVD event being considered as failure, or onset of diabetes, death or end of follow-up, defined as the last time point when all data and glucose measures were updated for each participant, or December 2009, whichever of them occurred first being considered as censoring event.

### Statistics

FPG, 1 hPG, 2 hPG and HbA1c were separately analyzed in relation to CVD incidence, adjusting for potential confounders. Stepwise adjustment for potential confounders was performed, initially for age, sex and smoking (Model 1), then adding body mass index (BMI) and physical activity (Model 2), and further adding systolic blood pressure (BP), low density (LDL) and high density (HDL) lipoprotein cholesterol (Model 3). The final model was additionally adjusted for history of CVD and cancer (Model 4). A sensitivity analysis was performed to determine whether group allocation or insulin resistance, measured by fasting serum insulin, contributed to risk of CVD. An additional sensitivity analysis was performed to include time and CVD events after diabetes onset by the 4 glycaemic measures, including the additional follow-up period at diabetes onset. A subgroup analysis investigating relation between FPG, 1 hPG, 2 hPG and HbA1c and the CVD incidence has been performed on patients with impaired fasting glucose (IFG), defined as FPG greater or equal to 5.6 mmol/l, at baseline (by definition, all of them had also IGT).

Semi-parametric Cox regression analysis was used to study the relation of the four glycaemic measures to CVD incidence. This method does not use any restriction on the baseline hazard function. The proportional hazards assumption was tested by including interaction terms for each covariate with the log(time) and was fulfilled for all glycaemic measures [10]. Potential confounders for the risk of CVD were included as baseline variables in a step-wise procedure (described above). Hazard ratios (HR) were estimated for each unit increase in the glycaemic measures. For the updated means and updated values, the HR per 1 unit increase in standard deviation (SD) was also estimated, assuming a normal distribution before estimating each HR. Updated mean and updated values of glycaemic measures having significant associations with CVD incidence were also analyzed pairwise in additive Cox models, as well as including all 4 main glycaemic variables at the same time, adjusting for all potential confounders.

## Results

### Characteristics of the cohort

Baseline characteristics are shown in Table 1. The mean age at baseline was 55.2 years, 32·9% were men, mean BMI was 31.3 kg/m^2^, and 6.0% were active smokers. Mean baseline FPG was 6.1 mmol/l, 1 hPG was 11.3 mmol/l, 2 hPG was 8.9 mmol/l and HbA1c was 5.6% (37.7 mmol/mol).

**Table 1:** Baseline characteristics of the study cohort.

	DPS CVD (n = 504)
Age (years) at Baseline	55.2 (7.1)
	57.0 (39.5; 67.9)
	n = 504
Gender	
Male	166 (32.9%)
Female	338 (67.1%)
Smoking at Baseline	
Non-smoker	474 (94.0%)
Smoker	30 (6.0%)
BMI (kg/m2) at Baseline	31.3 (4.5)
	30.4 (23.5; 50.5)
	n = 504
Leisure-time physical activity at Baseline	
None	174 (34.6%)
< = 4 h per Week	277 (55.1%)
>4 h per Week	52 (10.3%)
SBP (mmHg) at Baseline	138.1 (17.7)
	135.0 (98.0; 200.0)
	n = 504
HDL (mmol/L) at Baseline	1.21 (0.29)
	1.18 (0.59; 2.56)
	n = 503
LDL (mmol/L) at Baseline	3.61 (0.82)
	3.60 (1.68; 6.30)
	n = 501
Previous CVD Events	
No	489 (97.0%)
Yes	15 (3.0%)
Previous Cancer	
No	487 (96.6%)
Yes	17 (3.4%)
Fasting Plasma Glucose (mmol/l) at Baseline	6.14 (0.76)
	6.11 (3.73; 8.78)
	n = 504
One-hour Plasma Glucose (mmol/l) at Baseline	11.3 (2.3)
	11.2 (4.5; 18.6)
	n = 498
Two-hour Plasma Glucose (mmol/l) at Baseline	8.89 (1.49)
	8.72 (4.70; 13.71)
	n = 504
HbA1c (%) at Baseline	5.63 (0.57)
	5.60 (3.60; 8.00)
	n = 502

For categorical variables n (%) is presented.

For continuous variables Mean (SD)/Median (Min; Max)/n = is presented.

The composite CVD endpoint occurred in 34 (6.7%) individuals over a median follow-up period of 9.0 years, 8.0 events per 1000 patient-years, in participants during the prediabetic phase. When the length of follow-up after diabetes onset was also included, there were 52 (10.3%) composite endpoints over a median follow-up of 13.0 years, 9.3 events per 1000 patient-years.

**Figure 1:**
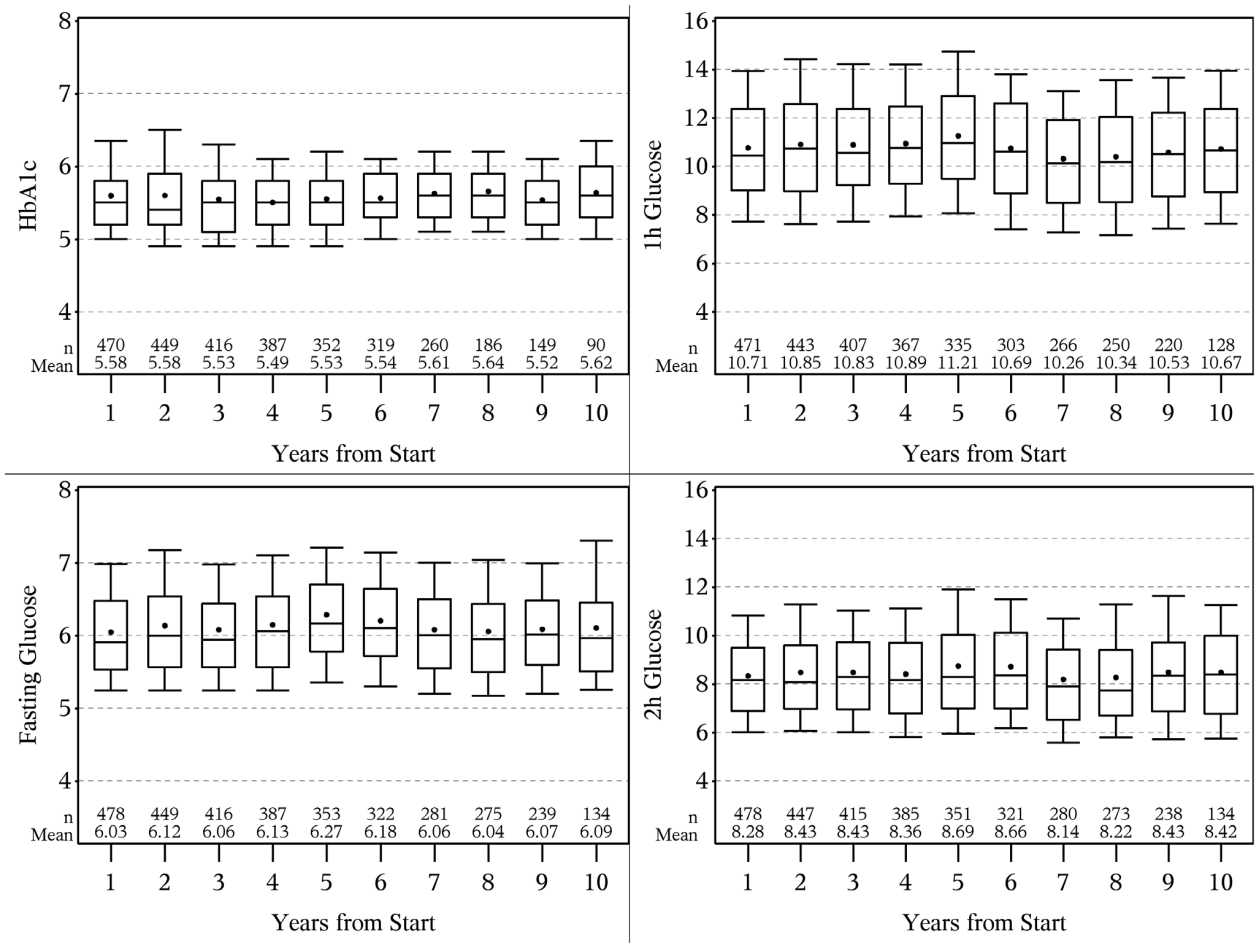
Mean level and number of measurements of FPG, HbA1c, 1-hour OGTT and 2-h OGTT during the 10-year follow-up.

Figure 1 shows the mean level and number of measurements of FPG, 1 hPG, 2 hPG and HbA1c for participants who did not develop diabetes during the first 10 years of follow-up. Mean FPG levels ranged between 6.0–6.3 mmol/l, 1 hPG from 10.3–11.2, 2 hPG from 8.1–8.7 and HbA1c from 5.5%–5.6% (36.6–37.7 mmol/mol).

### Updated mean and updated value of FPG, 1 hPG, 2 hPG and HbA1c

Relative risks of CVD events for the four glycaemic measures were estimated in stepwise increasing covariate models (Table 2). Updated mean and updated value of FPG was not associated with the incidence of CVD events in any of the four models (Table 2). There was a significant direct association between 1 hPG as well as 2 hPG and incidence of CVD events in all four models for both updated mean and updated values of these measurements. There was a direct and statistically significant relation between higher updated mean/updated value of HbA1c and incidence of CVD events.

**Table 2:** Hazard ratios of CVD for 1 unit increase in FPG, 1 hPG, 2 hPG and HbA1c.

	Hazard Ratio (95% CI) P-value			
	Model 1	Model 2	Model 3	Model 4
Updated mean value				
Mean Fasting Plasma Glucose (mmol/l)	1.67 (0.92−3.03)p = 0.09	1.53 (0.82−2.85)p = 0.18	1.60 (0.85−3.00)p = 0.14	1.66 (0.89−3.12)p = 0.11
Mean One-hour Plasma Glucose (mmol/l)	1.24 (1.02−1.50)p = 0.029	1.22 (1.00−1.48)p = 0.048	1.23 (1.00−1.50)p = 0.047	1.24 (1.01−1.52)p = 0.036
Mean Two-hour Plasma Glucose (mmol/l)	1.52 (1.16−2.01)p = 0.0027	1.48 (1.11−1.97)p = 0.0068	1.43 (1.07−1.90)p = 0.015	1.44 (1.08−1.92)p = 0.014
Mean HbA1c (%)	2.35 (1.24−4.46)p = 0.0088	2.23 (1.17−4.27)p = 0.015	2.29 (1.19−4.42)p = 0.013	2.73 (1.40−5.32)p = 0.0032
Updated value (last value prior to the CVD event)				
Fasting Plasma Glucose (mmol/l)	1.35 (0.82−2.20)p = 0.24	1.25 (0.76−2.08)p = 0.38	1.27 (0.76−2.11)p = 0.37	1.31 (0.78−2.19)p = 0.31
One-hour Plasma Glucose (mmol/l)	1.20 (1.03−1.40)p = 0.020	1.18 (1.01−1.38)p = 0.033	1.18 (1.01−1.39)p = 0.039	1.19 (1.02−1.40)p = 0.03
Two-hour Plasma Glucose (mmol/l)	1.47 (1.24−1.74)p = <.0001	1.46 (1.23−1.73)p = <.0001	1.44 (1.21−1.71)p = <.0001	1.45 (1.22−1.72)p = <.0001
HbA1c (%)	1.82 (1.05−3.16)p = 0.033	1.75 (1.01−3.06)p = 0.048	1.79 (1.02−3.15)p = 0.044	1.98 (1.11−3.51)p = 0.020
Baseline value				
Fasting Plasma Glucose (mmol/l)	1.53 (0.93−2.51)p = 0.097	1.42 (0.84−2.41)p = 0.19	1.53 (0.90−2.58)p = 0.11	1.61 (0.95−2.72)p = 0.077
One-hour Plasma Glucose (mmol/l)	1.18 (1.00−1.38)p = 0.0502	1.17 (0.98−1.38)p = 0.075	1.17 (0.98−1.40)p = 0.078	1.18 (0.99−1.41)p = 0.068
Two-hour Plasma Glucose (mmol/l)	1.25 (1.00−1.55)p = 0.052	1.20 (0.96−1.52)p = 0.11	1.16 (0.92−1.47)p = 0.21	1.16 (0.92−1.47)p = 0.22
HbA1c (%)	1.67 (0.91−3.09)p = 0.10	1.55 (0.83−2.89)p = 0.17	1.58 (0.85−2.96)p = 0.15	1.76 (0.92−3.36)p = 0.087

Model 1 Covariates: Age, Gender, Smoking.

Model 2 Covariates: Age, Gender, Smoking, BMI, Physical Activity.

Model 3 Covariates: Age, Gender, Smoking, BMI, Physical Activity, SBP, HDLC, LDLC.

Model 4 Covariates: Age, Gender, Smoking, BMI, Physical Activity, SBP, HDLC, LDLC, Previous CVD, Previous Cancer.

When updated means of 1 hPG and 2 hPG were included pairwise in the same Cox regression model adjusting for all possible confounders, the HRs were 1.14 (0.90–1.44), p = 0.28 for 1 hPG and 1.29 (0.93–1.80), p = 0.12 for 2 hPG. The HR for updated mean of 1 hPG was 1.13 (0.90–1.41), p = 0.29 and 2.19 (1.03–4.69), p = 0.043 for updated mean of HbA1c when included pairwise in the model. The HRs for updated mean 2 hPG and HbA1c were 1.30 (0.95–1.76), p = 0.10 and 2.22 (1.08–4.59), p = 0.031, respectively, when included pairwise in the model.

When updated 1 hPG and 2 hPG values were included pairwise in the same Cox regression model adjusting for all possible confounders, the HRs were 1.00 (0.82–1.21), p = 0.99 for 1 hPG and 1.45 (1.17–1.79), p = 0.0006 for 2 hPG. The HRs for updated 1 hPG and updated HbA1c were 1.14 (0.96–1.35), p = 0.13 and 1.65 (0.89–3.06), p = 0.11, respectively, and for 2 hPG and HbA1c were 1.40 (1.17–1.68), p = 0.0002 and 1.49 (0.81–2.74), p = 0.20, respectively, when included pairwise in the model.

When all four glycaemic variables were included as time-updated values in the same model including all possible confounders, the only glycaemic predictor that remained statistically significant was 2 hPG with HR of 1.44 (1.16–1.78), p = 0.0009.

### Baseline value of the four glucose measures

HRs for higher baseline values of the four glycaemic measures were greater than one in all models, although in all four models the association between each of the four measures and the incidence of CVD was not statistically significant (Table 2).

### Gradient of risk per 1- unit SD increase for updated mean and updated value of 1 hPG, 2 hPG and HbA1c

Since the updated mean values and updated values of 1 hPG, 2 hPG and HbA1c were significantly related to CVD, we standardised the risk gradients by estimating the HRs per 1 SD increment for each glycaemic measure. HRs estimates for the updated mean of 1 hPG, 2 hPG and HbA1c ranged from 1.45–1.51, 1.59–1.73 and 1.43–1.57 per 1-unit increase in the SD, respectively, in the four models (Table 3). For the updated values of 1 hPG, 2 hPG and HbA1c the HRs ranged from 1.51–1.57, 2.17–2.28 and 1.36–1.45 per 1-unit increase in the SD, respectively.

**Table 3:** Updated mean and updated value of FPG, 1 hPG, 2 hPG and HbA1c in relation to CVD by 1 unit higher SD of the variable.

	Hazard Ratio (95% CI) P-value			
	Model 1	Model 2	Model 3	Model 4
Updated mean value				
Mean Fasting Plasma Glucose	1.35 (0.95−1.93)p = 0.090	1.29 (0.89−1.86)p = 0.18	1.32 (0.91−1.91)p = 0.14	1.35 (0.93−1.96)p = 0.11
Updated Mean One-hour Plasma Glucose	1.50 (1.04−2.17)p = 0.029	1.45 (1.00−2.11)p = 0.048	1.48 (1.00−2.18)p = 0.047	1.51 (1.03−2.23)p = 0.036
Updated Mean Two-hour Plasma Glucose	1.73 (1.21−2.48)p = 0.0027	1.67 (1.15−2.42)p = 0.0068	1.59 (1.09−2.31)p = 0.015	1.60 (1.10−2.34)p = 0.014
Updated Mean HbA1c	1.47 (1.10−1.95)p = 0.0088	1.43 (1.07−1.92)p = 0.015	1.45 (1.08−1.95)p = 0.013	1.57 (1.16−2.11)p = 0.0032
Updated value (last value)				
Updated Fasting Plasma Glucose	1.25 (0.86−1.82)p = 0.24	1.19 (0.81−1.74)p = 0.38	1.20 (0.81−1.76)p = 0.37	1.22 (0.83−1.81)p = 0.31
Updated One-hour Plasma Glucose	1.57 (1.07−2.28)p = 0.020	1.51 (1.03−2.22)p = 0.033	1.52 (1.02−2.25)p = 0.039	1.55 (1.04−2.29)p = 0.030
Updated Two-hour Plasma Glucose	2.28 (1.59−3.26)p = <.0001	2.23 (1.55−3.21)p = <.0001	2.17 (1.50−3.14)p = <.0001	2.19 (1.51−3.18)p = <.0001
Updated HbA1c	1.39 (1.03−1.87)p = 0.033	1.36 (1.00−1.84)p = 0.048	1.37 (1.01−1.87)p = 0.044	1.45 (1.06−1.98)p = 0.020

Model 1 Covariates: Age, Gender, Smoking.

Model 2 Covariates: Age, Gender, Smoking, BMI, Physical Activity.

Model 3 Covariates: Age, Gender, Smoking, BMI, Physical Activity, SBP, HDLC, LDLC.

Model 4 Covariates: Age, Gender, Smoking, BMI, Physical Activity, SBP, HDLC, LDLC, Previous CVD, Previous Cancer.

### Sensitivity analysis

Neither the group allocation nor insulin resistance showed any significant association to the risk of CVD.

There were 239 individuals in the entire cohort who developed diabetes during the entire study period. In the sensitivity analysis including time after diabetes onset, the HRs for the updated means of FPG, 1 hPG, 2 hPG and HbA1c were 1.25 (0.85–1.83), p = 0.26, 1.17 (1.03–1.34), p = 0.018, 1.16 (0.99–1.35), p = 0.061 and 1.57 (0.94–2.60), p = 0.082, respectively. For updated values the corresponding HRs were 1.20 (0.94–1.53), p = 0.15, 1.12 (1.01–1.23), p = 0.027, 1.13 (1.04–1.23), p = 0.0031 and 1.43 (0.99–2.08), p = 0.059 for FPG, 1 hPG, 2 hPG and HbA1c, respectively.

### Subgroup analysis of patients with impaired fasting glucose

A subgroup analysis was performed in patients with impaired fasting glucose (76% of all patients). Unlike the results obtained in the main analyses, updated mean and baseline FPG were statistically significantly associated with the incidence of CVD events in all 4 models, with HR (95% CI) 2.37 (1.08−5.17), p = 0.031, and 2.22 (1.05−4.70), p = 0.038, respectively in model 4. In the analyses of last updated value the only significant predictor was 2 hPG with HR from model 4 being 1.52 (1.21−1.91), p = 0.0004. Similarly, the sensitivity analyses on this subgroup of patients showed only significant association between updated last value of 2 hPG and the incidence of CVD, 1.13 (1.03−1.24), p = 0.0093.

## Discussion

These new data from the DPS are the first to evaluate the association between glycaemic level and CVD incidence during a prediabetes phase by censoring patients with later development of diabetes based on annual repeated OGTTs. Thus this study is also the first prospective study with systematically repeated assessment of glycaemic values during the follow-up phase among prediabetic individuals. An increased level of the current 2 hPG, recorded as the updated (last) value of yearly estimates of 2 hPG during follow-up, was related to increased risk of CVD independent of other glycaemic markers such as FPG, 1 hPG and HbA1c in all models used to control effects of other variables. The current level of 2 hPG at the last visit before the CVD event also showed the highest risk increase for CVD (119%) when the various glucose measures were standardised to 1 SD increment. The current level of 2 hPG was also strongly associated with increased risk of CVD in sensitivity analyses including the follow-up time after diabetes diagnosis and in a subgroup analysis including patients with both IFG and IGT. Higher mean level of historical HbA1c-values was also independently related to an increased risk of CVD during the prediabetic phase, but this was not confirmed in sensitivity analyses including follow-up time after diabetes onset. An increased level of FPG was not associated with higher risk of CVD in any models of the study population but an association was found in the subgroup analysis of patients with IFG at baseline. 1 hPG was associated with increased risk of CVD in some models when included as the only glucose measure, but not independently when combined with other glucose measures in the same model. Since baseline values of glucose measures were generally poor predictors of CVD for all glucose measures, it raises the question of the validity of prospective studies where baseline data only have been available.

There are several previous studies using a single baseline value for glucose measures in predicting CVD or mortality but did not have information on later time measures after onset of diabetes [2]. This may be due to the fact that regular screening is needed to determine the true time of onset for diabetes, thus requiring greater resources. In such studies 2 hPG has generally been a stronger predictor than FPG for CVD and mortality [2], [6], [11]. A number of studies have also compared the predictive ability of FPG, 2 hPG and HbA1c for CVD or mortality in the same study population [12]–[16]. In the Rancho Bernardo Study [12], HbA1c was found to be a better predictor of CVD and ischaemic heart disease mortality than FPG or 2 hPG in women only. In contrast, other studies found that post-load 2 hPG was a better predictor of mortality and/or CVD outcomes than FPG or HbA1c [13]–[15]. In a study by Barr and colleagues [16], 2 hPG and FPG, but not HbA1c, were significant predictors of all-cause mortality, whereas all measures were significant predictors of CVD mortality. In a population-based prospective study among Finnish middle-aged people with IGT at baseline, those who had started with antidiabetic drug treatment during 10-year follow-up were identified [17]. The HRs for CHD incidence and mortality did not differ between those who had and had not developed diabetes before the CVD event. Monnier and colleagues showed that when HbA1c is below 7.3%, the contribution of postprandial, not fasting, glucose level is predominant [18], which has however been challenged in other studies [19]. The increased glucose level has also been associated with the CVD mortality in the newly diagnosed T2D patients [20].

The risk gradient in the current study of the updated value of 2 hPG was strong when compared with previous studies including people with prediabetes [2], [12], [16]. It was also strong when compared with relations observed between HbA1c and CVD among T2D patients where 1 SD higher HbA1c has been associated with a 25% increased risk of MI [9]. The current value of 2 hPG showed the strongest association with CVD, with a 119% increased risk per 1-unit higher SD 2 hPG and 51% increased risk if the lower boundary of the 95% CI is true. Such strong associations to CVD during the prediabetes phase may be due to our use of repeated measurements and updated recent values of glycaemia instead of using only baseline values. The general lack of relationships between baseline values of various glucose measures and risk of CVD in the present analysis also supports this theory and is potentially due to baseline values being poor surrogate markers for overall glycaemic control during follow-up, thereby weakening the association with CVD, known in epidemiology as regression dilution bias.

Debate continues over the type of glycaemic measure used to screen for prediabetes. We have previously shown that monitoring of HbA1c in prediabetic individuals is insensitive for the detection of new cases of T2D [21], [22]. Having the opportunity in this study to analyse the importance of the current level of various glucose measures, rather than using a baseline value several years old which is often the case for baseline values, our results indicate that the 2 hPG level is superior to other glucose measures including HbA1c in predicting CVD. Hence, the current analysis supports using OGTT as one tool in screening for prediabetes and to monitor when treating patients with prediabetes. HbA1c is probably also of predictive value for CVD when using the mean level of repeated measurements and, therefore, of potential use when following patients with existing prediabetes.

In the DECODE data there was no indication that a single measure of FPG is of any use for the prediction of CVD risk at the population level [6], [11]. The only study in the population with isolated IFG was negative [23]. Measurement of FPG for the assessment of CVD risk has be discouraged by recommendations provided in the current European guideline on Diabetes, Prediabetes and Cardiovascular Disease [24]. In this study FPG had no prognostic value of CVD when analyzing the whole population in this study comprised by patients of IGT. However, in a subgroup analysis of patients with IFG at baseline (76% of included patients) there was an association between both baseline and updated mean FPG with CVD. These results must however be interpreted with caution since they were derived from a subgroup analysis and the p-values were at the level 0.03–0.04.

Results from the Malmö Feasibility Study, a non-randomised study, and the Da Qing Study suggested that CVD rates might be reduced in individuals with IGT who participated in lifestyle intervention programmes [5], [25]. The STOP-NIDDM trial, including relatively few CVD cases, using acarbose as the intervention in individuals with IGT showed a statistically significant reduction in CVD rates [26]. Thus our results, that post-challenge glucose was significantly associated with CVD risk in DPS participants with IGT at baseline, are consistent with these earlier findings. Unfortunately, none of these studies were designed or powered to test the hypothesis of either therapy preventing CVD in individuals with IGT. Also, our comparison using the DPS cohort with the population-based cohort of Finnish people with IGT and of the same age as the Malmö Feasibility Study showed that total mortality and CVD risk were markedly lower in the DPS cohort irrespective of the intervention group [27].

A limitation of the present study is the relatively small number of patients and CVD events, (504 patients, 34 CVD events during the prediabetic phase and a total of 52 events after the diagnosis of diabetes). This implies that confidence intervals for the estimated HRs are relatively wide. On the other hand, one major and unique strength of this study is the repeated yearly recordings of FPG, 1 hPG, 2 hPG and HbA1c over a long period of time, information that is generally lacking in other studies due to many logistical reasons. HbA1c has previously been associated with CVD in people free of diabetes at baseline [28]. The current study should not be regarded as disqualifying HbA1c as a screening tool for diabetes, but rather showing additional benefits of 2 hPG over and above HbA1c. Therefore 2 hPG has an essential role in screening and monitoring prediabetic individuals. Similar methodology as in previous studies of type 2 diabetes relating updated mean HbA1c and baseline HbA1c to CVD events was used [29]. It should be noticed that evaluations of changes in risk by 1 SD higher of the risk factor used here to compare the predictive power of the various glucose measures may differ to some extent in other populations if the HbA1c distribution differs relative to the glucose measures’ distributions. However, it seems unlikely that the glucose pattern at a certain Hba1c-level should be substantially different in another population, especially if patients are not treated with glucose-lowering drugs. Moreover, also the fact that updated 2 hPG remained significant but updated HbA1c did not in pairwise comparisons and 2 hPG generally showing lower p-values in evaluated models supported its superiority as predictor.

In conclusion, this study demonstrates that level of glycaemic control already existing in the prediabetic phase is strongly related to risk of CVD. The current value of 2 hPG is the strongest predictor for CVD and should be one tool in screening for prediabetes and when monitoring the glycaemic control in patients with prediabetes. Prospective studies are needed to test and confirm these results from the DPS.
